# Developing and Evaluating an Educational Program for Respiratory Infection Prevention among Rural Elderly Residents in South Korea

**DOI:** 10.3390/ijerph17093057

**Published:** 2020-04-28

**Authors:** Jin Soon Kim, Ji Hye Choi, Myung Soon Kwon

**Affiliations:** 1 Dang-Rim Primary Health Care Post, Chuncheon Public Health Center, Chuncheon-si, Gangwon-do 24463, Korea; 2 Health and New Media Research Institute, Hallym University, Chuncheon-si, Gangwon-do 24252, Korea; 3 School of Nursing, Research Institute of Nursing Science, Hallym University, Chuncheon-si, Gangwon-do 24252, Korea

**Keywords:** educational program, respiratory infection prevention, rural elderly residents, social cognitive theory

## Abstract

Based on social cognitive theory (SCT), an educational program was developed to prevent rural elderly residents from respiratory infections in South Korea. The effectiveness of the program was investigated in terms of knowledge, attitudes, and practices about respiratory infection prevention, as well as social capital. A pretest–posttest nonequivalent control group quasi-experimental design was used to test the short-term effect of this program. In addition, 1- and 6-month follow-up surveys were administered to evaluate the long-term effects. A total of 69 subjects (37 in the experimental group and 32 in the control group) participated in the experiment. The results showed that knowledge about respiratory infection prevention, respiratory infection prevention practices, and social capital were enhanced among the elderly residents who participated in the educational program. The educational effects differed significantly across time periods (pretest, posttest, 1- and 6-month follow up) in all the above variables. In particular, the program remained effective 1 month after the intervention, but a reinforcement session extended the program’s effects up to 6 months later. This educational program would be used as an effective intervention to help rural elderly residents prevent respiratory infections.

## 1. Introduction

As a country whose aging population is growing faster than anywhere else in the world, South Korea is estimated to become a super-aged society—where 20 percent or more of the population is 65 or older—in 2025 and to have the largest elderly population worldwide by 2045 [1,2]. Since medical expenditure for the elderly constitutes nearly 40 percent (39.9%) of South Korea’s total medical expenditure [3], health management for the elderly population is an urgent issue in the country. In particular, almost one-half (44.7%) of the rural population is over 65 years of age [4]. Thus, health education and health promotion programs for rural elderly residents are needed to improve the quality of life of the elderly in South Korea.

Aging has been shown to increase the prevalence and severity of diseases, as well as the risk of mortality. According to Korean cause of death statistics, in 2018, the mortality rate from pneumonia was only 0.1 per 100,000 for 10–19 year olds, but increased rapidly with aging to 24.9 per 100,000 for 60–69 year olds, 144.0 per 100,000 for 70–79 year olds, and 978.3 per 100,000 for 80 years or older [5]. Medical costs of pneumonia patients over 65 years of age are 6.7 times higher than those of pneumonia patients aged 15 to 44 years, and three-quarters of these medical costs are paid for hospitalization [6]. Health risks associated with aging are caused by declining physical functioning, increased social isolation, greater risk of depression, and lowered resistance to the causative agents of infectious diseases, such as pneumonia and influenza, which affect the upper or lower respiratory tract. Such infections are especially risky for the elderly [7], representing a leading cause of death in this population. In fact, the mortality rate from pneumonia has increased over the past 10 years [3], with the highest rates occurring in Japan followed by South Korea [8]. It is therefore crucial to develop and implement an effective strategy to protect the elderly from respiratory infections.

The World Health Organization (WHO) has recommended both pharmacological interventions and non-drug treatments for the prevention of infectious diseases [9]. However, one major pharmacological intervention, vaccination, is often less effective among the elderly because of weakened immune systems, chronic disease complications, nutritional deficiencies, and lack of exercise, among other factors. In fact, the immunogenicity of vaccines among the elderly is in the range of 30–40%, compared to 70–90% for healthy adults [10,11]. Thus, non-drug preventive methods, such as personal hygiene practices (e.g., hand washing and respiratory hygiene) and improved healthy lifestyle practices (e.g., healthier diets, exercise, oral hygiene, sleep, and rest), are also recommended for the prevention of respiratory infections [12,13]. In addition, optimal indoor ventilation, temperature, and humidity are important safeguards, as is the observation of cough etiquette [14].

Since 1981, South Korea has established public health facilities and assigned dedicated healthcare workers, not only to improve access to healthcare services, but also to provide comprehensive primary healthcare services for medically vulnerable rural residents. Rural healthcare providers are generally local government officials who have nursing or midwifery licenses. These officials are assigned to public health facilities to conduct medical activities and health promotion among rural residents [15]. Therefore, an educational approach to the prevention of infections may be among the most effective healthcare methods, especially in areas where the elderly population are in the majority, such as rural towns and villages. This is because rural elderly residents generally experience a lack of information on how to prevent respiratory infections, and other routes (e.g., physician–patient communication, Internet) for obtaining such information are very limited. Previous studies related to respiratory infection prevention, however, have typically only been conducted to examine the knowledge, attitudes, and practices concerning hand washing and cough etiquette among urban elderly populations [16]—and as such, resulting respiratory infection prevention programs have been developed primarily for urban elderly residents [17]. No such prevention program has been developed for rural elderly residents.

Based on Bandura’s social cognitive theory (SCT) [18], which can infer learning motives through observation and experience, we developed an educational program to effectively convey knowledge about infectious disease prevention to the elderly. SCT argues that human beings, as social agents, learn and adopt desirable behaviors not only from the direct experience of reward and punishment but also from vicarious experiences, i.e., observing the behaviors of others, as well as the consequences of these behaviors. This is called observational learning. According to Bandura, observational learning occurs via four component processes: (1) attention, (2) memory, (3) retention, and (4) motivation [18]. More specifically, attention represents the basic step by which individuals learn behaviors and their consequences from role models. Memory is the cognitive mechanism by which observers reflect on these behaviors and consequences. At this stage, most observed behaviors are memorized verbally, but others are memorized through imagery, primarily when verbal expressions are not available. The retention stage is the process through which behaviors stored in memory are directly reconstructed and expressed in an observer’s actions. The final step, motivation, is the process through which an observer’s desires are used to motivate real action. Although observers can accurately remember the behaviors of role models, without a corresponding motivation to act on these observed behaviors, the observer’s behavior will not change. In this study, the principle of observational learning of SCT was used to develop the educational program to enhance the participants’ attention to respiratory infection prevention and to strengthen their motivation for respiratory infection prevention practices in a real-life context.

In light of this, we aimed to develop a theory-driven health education program for the prevention of respiratory infections among elderly residents of rural areas. In addition, we aimed to evaluate the effectiveness of the program among the rural elderly residents. Guided by the knowledge–attitude–practice (KAP) model, the effects of the program were investigated with knowledge, attitudes, and practices concerning respiratory infection prevention [19], together with social capital in the rural context. Thus, this study was guided by three research questions:

Research Question 1. Are there any differences in knowledge about respiratory infection prevention, attitudes toward respiratory infection prevention, respiratory infection prevention practices, and social capital between experimental and control groups?

Research Question 2. Are there any differences in knowledge about respiratory infection prevention, attitudes toward respiratory infection prevention, respiratory infection prevention practices, and social capital across pretest, posttest, 1-month, and 6-month follow-ups?

Research Question 3. Do the experimental-control group differences vary across pretest, posttest, 1-month, and 6-month follow-ups?

## 2. Materials and Methods

### 2.1. Study Design and Participants 

Based on Bandura’s SCT [18], a health education program aimed at preventing respiratory infections among rural elderly residents was developed, and the effects of this program were tested using a nonequivalent control group quasi-experimental design with both pretest and posttest (Table 1). This study was conducted under the oversight of the Hallym University Institutional Review Board (IRB), IRB # 2018-066-1-R.

The subjects of this study were rural elderly individuals (over 60 years of age) with no visual, auditory, cognitive, and/or speech impairments. Before the experiment began, all subjects were explained in detail the purpose and procedure of the study and the potential risks and benefits of participation afterward. A one-to-one explanation of the study was also provided to each subject and then the subjects were asked to sign a written consent form if they agreed to participate in the study. They understood the purpose of this study and consented to participation in the experiment. In order to select the public health facilities for research participation, the researcher contacted 13 public health facilities located outside the city, two of which showed an intention to participate in this study. Of those who were under the care of the two public health facilities, the elderly who indicated their intention to participate in this study were recruited as the subjects through the health facilities. One of the two public health facilities was selected for the experiment since this facility expressed interest in participating in the educational program. Therefore, elderly individuals under the care of this facility comprised the experimental group. The control group, on the other hand, comprised elderly individuals who were under the care of the other public health facility. Both public health facilities were in the same administrative district so that the elderly residents lived in similar rural settings. However, the two facilities were geographically far apart from each other; that is, one of the facilities was located on the south side of a river and the other on the north side.

**Figure 1:**
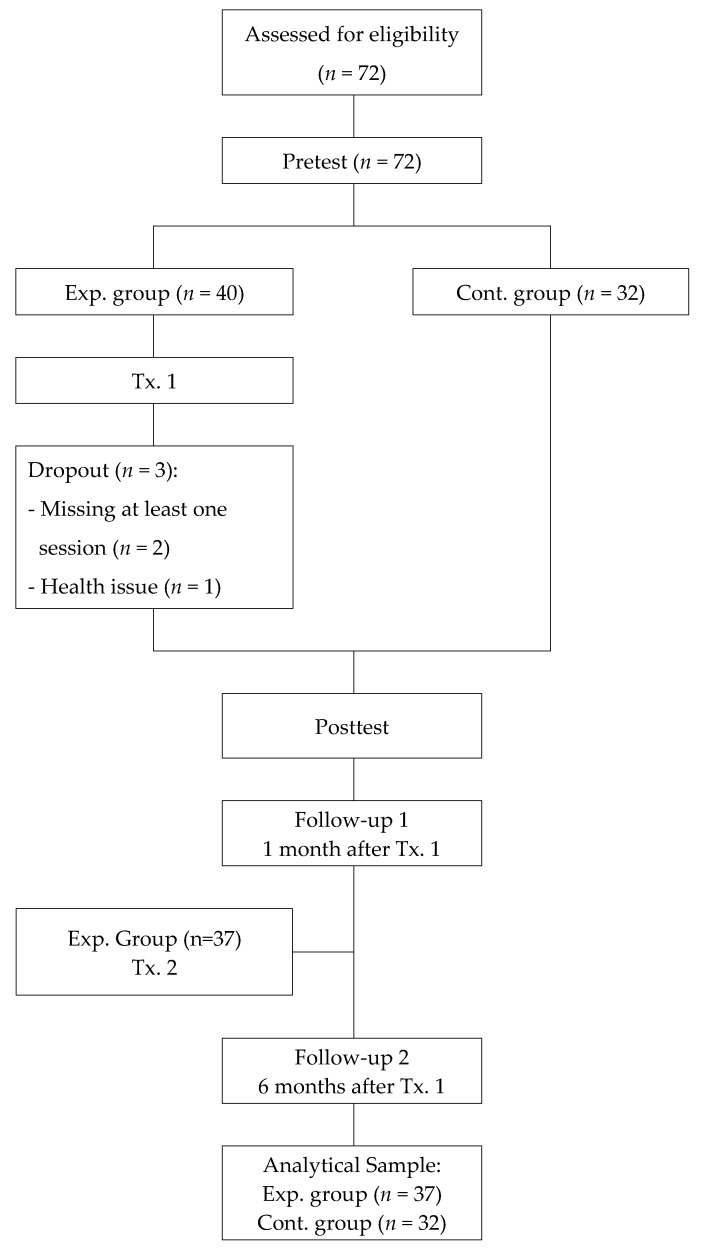
Flow chart of the study.

The sample size required for this study was calculated using G*power 3.1 (Heinrich Heine University, Düsseldorf, Germany), in which the values of significance level, effect size, and power were 0.05, 0.30, and 0.80, respectively, for repeated-measures analysis of variance (repeated ANOVA). The result showed that this study would require at least 58 subjects; thus, a total of 72 individuals were recruited to account for potential dropout. In fact, three people in the experimental group were excluded: two for missing one of the four training sessions, and one due to a health issue. Thus, the final analytical sample comprised 69 subjects, 37 in the experimental group and 32 in the control group (Figure 1). 

### 2.2. The Educational Program: Respiratory Infection Preventive Education Program based on Social Cognitive Theory (RIPEP-SCT)

The RIPEP-SCT was developed after reviewing a number of studies that addressed the prevention of respiratory infections [12,17], as well as theories used to explain effective educational methods among the elderly [20,21], including Bandura’s SCT [22,23,24]. Based on lessons learned from previous research and from SCT [18], the RIPEP-SCT was designed according to four observational learning processes: attention, memory, retention, and motivation.

The RIPEP-SCT comprised four training sessions held over the course of four weeks, with one session per week, each 50 min in duration. A reinforcement session was also held about one to six months after the initial training (Table 2). The educational program was applied to the residents of four town halls on each day of the week, and no more than 15 trainees were allowed to attend at a time. Before the program started, an introductory session was provided to the trainees, after which they were asked to create an attendance table and complete a pretest questionnaire. More specifically, the trainees were asked to take a photo and attach it to a large board on the wall of the town hall for the attendance table. They were also asked to write down the date of their participation on the attendance table and place a sticker directly on the table when they had completed participation in each session. This was purposely designed to help the trainees easily check their attendance. Afterwards, the purpose and schedule of the program were explained, and a face-to-face survey was conducted for the pretest.

During each training session, one topic was covered, such as symptoms of respiratory infections, proper cough etiquette, correct hand washing and dental hygiene, and healthy walking. The content of the sessions reflected the four observational learning processes. First, to reflect the stage of attention, the trainees were asked to discuss what they already knew about respiratory infections, and they were shown some visual stimuli depicting respiratory infections to enhance their awareness of the severity of these infections. The attentional level of each trainee was also determined by asking them about their experiences with the topics to be covered in the following session. From the memory stage to the motivation stage, lessons learned from the previous sessions were created as a song to make them easy to remember, and the trainees were asked to share their experiences with prevention practices. Reflecting the memory stage, the educational material was designed to stimulate the five primary senses. For example, visual stimuli (i.e., images and videos) were presented using PowerPoint to stimulate hearing and sight, and a dental model was provided to the trainees to stimulate sight and touch. The dental model was an actual model of teeth, and it was used to teach correct brushing techniques because oral hygiene is an important part of preventing respiratory infections. Looking at the model and touching it was intended to increase the educational effect of the elderly who experience a decline in cognitive function. Also, the trainees were asked to sing a song reflecting the melody of a happy birthday song, which allowed them to more easily remember the core educational content. They were also asked to recite the pledge of practice promoted in the sessions. For the retention stage, the program was constructed in the form of demonstration and experience. More specifically, the instructor showed the trainees how to properly wear a mask in the first training session. In the second training session, a demonstration was provided to the trainees to educate them about the six steps of correct hand washing. Next, the trainees were asked to wash their hands with soap and water. Their hands were checked through a hand hygiene training view box, which is designed to instantly highlight any germs on hands under UV light. In the third session, the instructor showed the trainees how to correctly brush their teeth, and they were given toothpaste and toothbrushes to use for practice. In addition, the trainees were asked to conduct an oral routine in front of a mirror after a demonstration was given by the instructor. Although physical activity is important for enhancing immunity, only 37.3% of the elderly in Korea meets the recommended physical activity guideline for more than 30 minutes a day and 5 times a week [25]. Therefore, correct walking means meeting physical activity standards and walking in the right posture in this study. In the fourth session, correct walking was demonstrated by the instructor, after which each trainee practiced correct walking. This activity was performed in front of the other trainees on a walking mat, which helped the trainees correct their walking posture either by themselves or through peer support and feedback. For the motivation stage, rewards were given to the trainees who practiced what they had learned in front of their peers. The instructor also gave rewards to trainees who excelled in each session. The trainees often sang songs together, collectively recited the pledge of practice, and took quizzes. Rewards (i.e., items for respiratory infection prevention such as a handkerchief, hand sanitizer, and toothbrush/toothpaste) were given to the trainees who answered questions correctly, which in turn increased their motivation to participate in the program. 

After the completion of all the sessions, a ceremony was held to celebrate their graduation from the program, and they were asked to complete the posttest questionnaire. During the ceremony, the trainees watched videos that had been recorded during the training sessions, and a certificate and prize were given to each of them. The trainees reflected on what they had learned during the training sessions by singing songs about each subject and reciting the pledge of practices. Lastly, time was allocated for a group discussion and evaluation of the program.

About one to six months after the program ended, a reinforcement session was provided to the trainees. In the reinforcement session, the trainees were asked to summarize the focal point of the four sessions and to remember the songs and the pledge of practice. A game format was employed to enhance their interest and motivation. Rewards were again used to motivate the participants to remember what they had learned (Table 2).

### 2.3. Measures

Knowledge about respiratory infection prevention includes the awareness and understanding of how to prevent these infections [26]. In this study, we used the respiratory infection prevention knowledge scale developed by Kwon and Yu [27]. The scale consists of 10 dichotomous items (i.e., yes/no questions), including two items for general knowledge, two items for symptoms and complications, one item for propagation path, two items for risk factors, and three items for preventive behaviors. In this study, the reliability of the scale was 0.69, which was obtained by the Kuder–Richardson Formula 20 (KR-20).

Attitudes toward respiratory infection prevention entail a mindset geared for preventing respiratory infections [26]. In this study, we used the respiratory infection prevention attitude scale developed by Kwon and Yu [27] to measure attitudes. The scale comprises 10 items assessed on a 5-point Likert-type scale (1 = strongly disagree, 5 = strongly agree), with one item for symptom management, two items for hand washing, one item for oral hygiene, two items for cough etiquette, one item for vaccination, two items for exercise and sleep (to increase resistance), and one item for participatory intention toward the program. In this study, the Cronbach’s alpha of the scale was 0.85.

Respiratory infection prevention practices encompass behaviors directly aimed at respiratory infection prevention [26]. In this study, the respiratory infection prevention attitude scale developed by Kwon and Yu [27] was used to measure these practices. This instrument employs 10 items assessed on 5-point Likert-type scale (1 = never, 5 = always), including one item for symptom management, two items for hand washing, one item for oral hygiene, two items for cough etiquette, one item for vaccination, two items for exercise and sleep (to increase resistance), and one item for participatory intention toward the program. In this study, the Cronbach’s alpha for the scale was 0.80.

Social capital was measured using the scale deployed by Shin and Ko [28]. A total of 12 items are included in the scale, along four dimensions, including three items for trust, three items for norms (reciprocity), three items for participation, and three items for networking. Trust refers to the degree of credibility one has in the eyes of others, while norms refer to often unwritten or unspoken social regulations and rules. Participation refers to involvement in community events. Networking refers to the degree and extent of interaction one has with family members, friends, and peers [28]. These items were assessed on a 5-point Likert-type scale (1 = not at all, 5 = extremely). The Cronbach’s alpha of the scale was 0.88 in this study.

All measures of this study were subjective, indicating that the term ‘knowledge’, ‘attitudes’, ‘practices’, and ‘social capital’ actually refers to perceived knowledge, perceived attitudes, perceived practices, and perceived social capital.

### 2.4. Procedure

The experiment was conducted in two public health facilities in rural areas. The experimental group comprised residents from four rural towns who were under the care of one public health facility; the control group comprised residents who were under the care of the other public health facility. The researchers explained the details (i.e., purpose, procedure, and method) of the research study and the rights of the participants. Before participation, each participant had to sign a consent form. Data were collected from December 4, 2018 to June 25, 2019 through a one-by-one, face-to-face survey. The participants were fully informed of their right to leave or withdraw from the study at any time for any reason. All participants were compensated with five-dollar gifts (e.g., hand sanitizer and hand creams, no slip socks, etc.) for their participation.

The educational program was conducted by community health experts who were certified as home and health nurses and had over 20 years of experience. They participated in the development of the program and worked as program instructors. In addition to the nurse instructors, four nursing students (three undergraduate students and one graduate student) were employed as research assistants for the experiment. Before the program began, a training session was provided to the nurses and research assistants to ensure that they fully understood the program procedures. After conducting a preliminary test, two researchers were asked to share the difficulties and problems they encountered and to provide feedback aimed at reducing research bias.

For the experiment, the participants were asked to complete a consent form after the researchers explained the purpose of the study. Subsequently, a face-to-face survey was conducted for the pretest. The experimental group received five training sessions of the educational program in the town hall, whereas the program was not applied to the control group. To evaluate the program, both experimental and control groups completed pretest, posttest, and one- and six-month follow-up surveys. A total of 72 subjects (40 in the experimental group and 32 in the control group) were initially recruited for the study, but three from the experimental group were ultimately excluded, as explained above. Thus, the final analytical sample comprised 69 individuals (37 in the experimental group and 32 in the control group). The training sessions were provided by nurse instructors, with assistance from six to seven researchers. The ages of the instructors were approximate to the ages of the trainees to promote instructor–trainee familiarity. The posttest was conducted immediately after the training sessions had ended for the experimental group. The control group completed posttest with no training. 

Both experimental and control groups completed two follow-up surveys 1 and 6 months after the program. In the meantime, a reinforcement session was provided to the trainees. In the reinforcement session, the trainees were asked to recall not only what they had learned during the four training sessions but also the songs they had sung as well as the pledge of practice. A game format was adopted to enhance the trainees’ interest in the program and increase their motivation to remember the focal point of the training sessions. 

### 2.5. Data Analysis

The questionnaire included questions about participants’ demographic characteristics, health-related attributes, and social capital, as well as their knowledge, attitudes, and practices concerning respiratory infection prevention. A chi-square test, Fisher’s exact test, and independent sample *t*-test were performed to determine intergroup homogeneity. A mixed analysis of variance (mixed ANOVA) was used to test the effectiveness of the educational program, since this type of analysis is likely to be more robust than other statistical methods when the assumption of homogeneity of variances is violated. To test the hypotheses, the mixed ANOVA was performed separately with four dependent variables (i.e., knowledge, attitudes, practices, and social capital) to investigate whether any significant differences existed between control and experimental group members concerning their knowledge, attitudes, and practices regarding respiratory infection prevention, as well as social capital across the pretest, post-test, one-month, and six-month follow-up assessments. Toward this end, one within-subjects factor (i.e., time: pretest, post-test, and both follow ups) and one between-subjects factor (i.e., group: control and experimental) were included in the data analysis. 

## 3. Results

### 3.1. Homogeneity Testing on Dependent Variables

The analytical sample comprised 69 individuals: 37 in the experimental group and 32 in the control group. The results of homogeneity tests showed no significant differences between the experimental and control groups in terms of knowledge (*t* = 0.90, *p* = 0.373), practices (*t* = −1.78, *p* = 0.080), and social capital (*t* = −0.97, *p* = 0.335), whereas a significant difference was found in attitudes (*t* = −2.70, *p* = 0.009) (Table 3).

### 3.2. Research Question 1: Differences between Groups

With regard to RQ1, four separate mixed ANOVAs were conducted to determine whether group differences existed in: (1) knowledge about respiratory infection prevention; (2) attitudes toward respiratory infection prevention; (3) respiratory infection prevention practices; and (4) social capital. 

For knowledge about respiratory infection prevention, the mixed ANOVA results indicated a statistically significant main effect of group, *F*(1, 67) = 7.34, *p* = 0.009, $\eta_{p}^{2}$ = 0.10 (Table 4). Specifically, the experimental group’s knowledge (*M* = 0.96, *SE* = 0.01) of respiratory infection prevention was better than that of the control group (*M* = 0.92, *SE* = 0.01). There was also a statistically significant main effect of group for respiratory infection prevention practices, *F*(1, 67) = 20.56, *p* < 0.001, $\eta_{p}^{2}$ = 0.24 (Table 4). The experimental group (*M* = 4.26, *SE* = 0.05) undertook more respiratory infection prevention practices than the control groups (*M* = 3.96, *SE* = 0.05). In terms of social capital, a statistically significant main effect of group was found in this study, *F*(1, 67) = 17.95, *p* < 0.001, $\eta_{p}^{2}$ = 0.21 (Table 4). The score of social capital in the experimental group (*M* = 4.16, *SE* = 0.05) was higher than that of the control group (*M* = 3.86, *SE* = 0.05). For attitudes toward respiratory infection prevention, however, there was no statistically significant main effect of group, *F*(1, 67) = 0.12, *p* = 0.734, $\eta_{p}^{2}$ = 0.00 (Table 4).

### 3.3. Research Question 2: Differences across Time Periods 

Regarding RQ2, four mixed ANOVAs were performed separately to test whether differences would be found across time periods for: (1) knowledge about respiratory infection prevention; (2) attitudes toward respiratory infection prevention; (3) respiratory infection prevention practices; and (4) social capital. 

For knowledge about respiratory infection prevention, the Huynh–Feldt correction was applied for non-sphericity. The mixed ANOVA results indicated a statistically significant main effect of time, Wilks’ lambda = 0.66, *F*(2.44, 163.38) = 9.50, *p* < 0.001, $\eta_{p}^{2}$ = 0.12 (Table 4). Pairwise comparison results showed statistically significant differences in the knowledge scores between pretest and posttest (*F*(1, 67) = 5.88, *p* = 0.018, $\eta_{p}^{2}$ = 0.08) and between posttest and 1-month follow-up (*F*(1, 67) = 10.99, *p* = 0.001, $\eta_{p}^{2}$ = 0.14), whereas no statistically significant difference was found in the knowledge scores between 1- and 6-month follow-ups (*F*(1, 67) = 2.94, *p* = 0.091, $\eta_{p}^{2}$ = 0.04). More specifically, the knowledge score was highest in the order of 1-month follow-up (*M* = 0.97, *SE* = 0.01), 6-month follow-up (*M* = 0.95, *SE* = 0.01), posttest (*M* = 0.94, *SE* = 0.01), and pretest (*M* = 0.90, *SE* = 0.02). 

Furthermore, a significant main effect of time was found for attitudes toward respiratory infection prevention, Wilks’ lambda = 0.65, *F*(3, 201) = 10.03, *p* < 0.001, $\eta_{p}^{2}$ = 0.13 (Table 4). Pairwise comparison results revealed that significant differences were found in the attitude scores between pretest and posttest (*F*(1, 67) = 5.90, *p* = 0.018, $\eta_{p}^{2}$ = 0.08) and between 1- and 6-month follow-ups (*F*(1, 67) = 21.65, *p* < 0.001, $\eta_{p}^{2}$ = 0.24), whereas there was no significant difference in the attitude scores between posttest and 1-month follow-up (*F*(1, 67) = 3.42, *p* = 0.069, $\eta_{p}^{2}$ = 0.05). The attitude score was highest in the order of 6-month follow-up (*M* = 4.85, *SE* = 0.03), posttest (*M* = 4.72, *SE* = 0.04), 1-month follow-up (*M* = 4.62, *SE* = 0.05), and pretest (*M* = 4.57, *SE* = 0.05). 

In terms of respiratory infection prevention practices, there was a statistically significant main effect of time, Wilks’ lambda = 0.59, *F*(2.81, 188.06) = 18.85, *p* < 0.001, $\eta_{p}^{2}$ = 0.22 (Table 4). The degrees of freedom were corrected using the Huynh–Feldt estimates since the assumption of sphericity was violated. Pairwise comparison results showed that there were significant differences in the practice scores between pretest and posttest (*F*(1, 67) = 35.11, *p* < 0.001, $\eta_{p}^{2}$ = 0.34), between posttest and 1-month follow-up (*F*(1, 67) = 10.61, *p* = 0.002, $\eta_{p}^{2}$ = 0.14), and between 1- and 6-month follow-ups (*F*(1, 67) = 10.52, *p* = 0.002, $\eta_{p}^{2}$ = 0.14). The practice score was highest in the order of 6-month follow-up (*M* = 4.28, *SE* = 0.04), posttest (*M* = 4.26, *SE* = 0.06), 1-month follow-up (*M* = 4.07, *SE* = 0.05), and pretest (*M* = 3.83, *SE* = 0.06).

In addition, the mixed ANOVA results showed that there was a statistically significant main effect of time for social capital, Wilks’ lambda = 0.91, *F*(2.60, 174.30) = 3.17, *p* = 0.032, $\eta_{p}^{2}$ = 0.05 (Table 4). The degrees of freedom were corrected using the Huynh–Feldt estimates because of non-sphericity. Pairwise comparison results revealed a significant difference in the social capital scores between pretest and posttest (*F*(1, 67) = 6.08, *p* = 0.016, $\eta_{p}^{2}$ = 0.08), whereas no significant difference was found between posttest and 1-month follow-up (*F*(1, 67) = 1.66, *p* = 0.202, $\eta_{p}^{2}$ = 0.02) and between 1- and 6-month follow-ups (*F*(1, 67) = 0.64, *p* = 0.427, $\eta_{p}^{2}$ = 0.01). The social capital score was highest in the order of posttest (*M* = 4.09, *SE* = 0.06), 6-month follow-up (*M* = 4.05, *SE* = 0.04), 1-month follow-up (*M* = 4.01, *SE* = 0.05), and pretest (*M* = 3.88, *SE* = 0.08).

### 3.4. Research Question 3: Interaction Effects between Group and Time

With regard to RQ3, four 2 × 4 mixed ANOVAs were performed separately with group (experimental and control groups) as a between-subjects factor and time (pretest, posttest, 1-month, and 6-month follow-ups) as a within-subjects factor on: (1) knowledge about respiratory infection prevention; (2) attitudes toward respiratory infection prevention; (3) respiratory infection prevention practices; and (4) social capital.

The mixed ANOVA revealed a significant interaction between group and time on the knowledge, Wilks’ lambda = 0.85, *F*(2.44, 163.38) = 3.09, *p* = 0.038, $\eta_{p}^{2}$ = 0.04. For attitudes toward respiratory infection prevention, there was a significant interaction between group and time, Wilks’ lambda = 0.75, *F*(3, 201) = 8.03, *p* < 0.001, $\eta_{p}^{2}$ = 0.11. In terms of respiratory infection prevention practices, a significant interaction between group and time was found in this study, Wilks’ lambda = 0.69, *F*(2.81, 188.06) = 14.10, *p* < 0.001, $\eta_{p}^{2}$ = 0.17. In addition to these three variables, there was also a significant interaction between group and time for social capital, Wilks’ lambda = 8.79, *F*(2.60, 174.30) = 10.81, *p* < 0.001, $\eta_{p}^{2}$ = 0.14 (Table 4).

**Figure 2:**
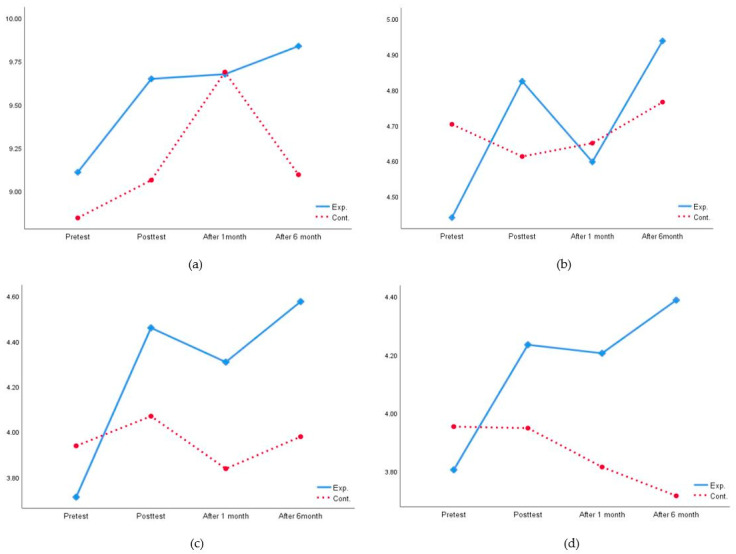
Interaction effects between group and time on: (**a**) knowledge about RIP; (**b**) attitudes toward RIP; (**c**) RIP practices; (**d**) social capital.

The means and standard deviations by group and time for the above four variables are shown in Table 4. Figure 2 graphically represents the interaction between group and time. Inspection of the figure suggested the experimental group had more knowledge about respiratory infection prevention at posttest and 6-month follow-up than did the control group; whereas the knowledge difference between the experimental and control groups was less pronounced at pretest and 1-month follow-up. For attitudes toward respiratory infection prevention, the control group had greater attitudes toward respiratory infection prevention than did the experimental group at pretest and 1-month follow-up, but not at posttest and 6-month follow-up. In regards to respiratory infection prevention practices, the experimental group was more likely to adopt respiratory infection prevention practices than was the control group at posttest, 1-month, and 6-month follow-ups, but not at pretest. A similar pattern was found in social capital. The experimental group reported higher social capital than did the control group at posttest, 1-month, and 6-month follow-ups, even though the control group reported higher social capital than did the experimental group at pretest.

## 4. Discussion

### 4.1. Development of a Health Education Program for Respiratory Infection Prevention

In this study, an educational program was developed to prevent respiratory infections among rural elderly people (over 60 years of age), and the effectiveness of the program was investigated. With the purpose of enhancing awareness among the elderly about respiratory infections, the educational program was designed on the basis of psychological, physical, and mental characteristics associated with old age [22]. The elderly are less likely to remember recent events than past events, and auditory information is more likely to be retained than visual information. Considering that depression increases in old age, strengthening social networks among the elderly may be effective for overcoming depression. Also, the elderly often experience changes in neurological and cognitive faculties, such as a slowing of reflexes and gradual desensitization. In addition to cognitive functions, concentration is also weakened in old age [29]. Therefore, we developed a health education program based on the processes of observational learning (i.e., attention, memory, retention, and motivation) derived from SCT [22]. Key concepts from this theory, such as imitation, cognition, reinforcement, and self-efficacy, were also applied to the development of the program. 

One of the four observational learning processes, attention, was incorporated in the program in such a way as to enhance trainees’ interests and motivation: the trainees were asked to think about why the topic of each training session was important. They were also asked to reflect on what they had learned by singing a song or taking a pledge of practice. The memory process, which involves the cognitive storage of information learned from observation, was promoted in the program through video materials, which were used to stimulate both vision and hearing among the trainees. To enhance their concentration, a variety of instruments were used, such as a dental model, a view box, masks, and a walking mat. These instruments helped to demonstrate actions like correct hand washing, correct walking, and so on. Next, the retention process, which involves determining the level of consistency between what one learns and how one subsequently behaves through internal feedback, was invoked among trainees through self-observation and self-correction, i.e., the trainees were guided to adapt their behavior to the model’s behavior. After demonstrations by the instructor, the trainees were instructed to use the program instruments to practice various activities, such as correct hand washing and correct toothbrushing, and they were also given opportunities for self-correction via feedback from the instructor or from their peers. As the final step, the motivation process was stimulated by encouraging desire among trainees to engage in program practices. The trainees sang a song that included keywords and key concepts from each training session. In addition, the trainees were also asked to take a pledge of practice to review what they had learned.

Rewards were used to increase participation among the trainees across all sessions. At the beginning of each session, rewards were given to the trainees who shared what they had learnt from the previous session with their peers. Some trainees were also rewarded with items for respiratory infection prevention such as a handkerchief, hand sanitizer, and toothbrush/toothpaste for completing tasks, memorizing the songs and pledge of practice, or correctly answering questions on the quiz. In the final session, certificates and prizes were awarded to the trainees for participating in the program.

For the program, instructors were selected based on whether their ages approximated those of the trainees, for the purpose of enhancing rapport and familiarity between them. Such an approach has previously been applied as an effective method for increasing compliance with respiratory infection prevention practices [30].

Similar to this study, many studies have used SCT to develop health education programs, such as a food safety education program for the elderly [23], a web-based hip fracture prevention program for the elderly [22], and an eye health program for children [24]. Based on SCT, these programs were constructed to reflect the characteristics and needs of the trainees, with the number of sessions typically ranging from 2–10 [23,30].

Unlike the research of Lee and Park [30] or Choi et al. [23], Nahm et al. [22] developed an online program in which learning materials were uploaded to a website. For this reason, only those who could access the Internet were able to participate in the study. In the study, the trainees were asked to study four learning modules for two weeks. In the meantime, assignments and discussions were posted on an online bulletin board, and the administrator checked the participation status of each trainee through their feedback or responses. Like other studies, the educational program used by Nahm and colleagues was designed to enhance trainees’ understanding via repetitive learning and experiences in which the characteristics of the trainees were reflected, such as educational levels and social support systems. A reinforcement session was also provided to the trainees between one and six months after the intervention for the purpose of evaluating its long-term effectiveness.

By combining the strengths of the existing programs, we developed a theory-driven intervention program aimed at educating rural residents about respiratory infection prevention. It was designed to facilitate behavioral changes among trainees, allowing them to more easily understand what they have learned from the program [31]. The effectiveness of the program was evaluated in both short-term and long-term follow-ups. 

### 4.2. Effectiveness of the Educational Program for Respiratory Infection Prevention

With the purpose of evaluating the effectiveness of the respiratory infection prevention program, this study examined the knowledge, attitudes, and practices concerning respiratory infection prevention among rural elderly residents, as well as their social capital, before and after the intervention. A reinforcement session was also provided about one to six months after the educational program ended. In this study, knowledge, attitude, and practice (KAP) of trainees were assessed because many studies have empirically examined these variables to evaluate the effectiveness of educational intervention [18,32]. In addition to the KAP, social capital was used as a key variable in this study because it often mediates the attitude–practice relationship [33] and affects the practice of health behaviors [34]. After a follow-up survey was conducted one month after the program ended, another 6-month follow-up survey was performed to investigate the long-term effects of the program, because six months is considered a sufficient timeframe for confirming whether behavioral changes have actually occurred [35]. 

In this study, all the research questions were supported, except for a group difference in attitudes. In the experimental group, knowledge about respiratory infection was enhanced by the educational program, peaking six months after the program ended. In contrast, knowledge among control group members increased by the 1-month follow-up, but then decreased. This may be because the participants in the control group were given the same questionnaire on three separate occasions, which may have consequently improved their knowledge, albeit slightly. In addition to knowledge differences, significant differences were found in attitudes across time periods. This effect was qualified by a significant interaction with the group. In terms of respiratory infection prevention practices, significant differences were observed between groups and across time periods. The experimental group was consistently high in practice at all three time points of the investigation (i.e., immediately after, one month after, and six months after the intervention), whereas practices among control group members were reduced one month after the program ended. The findings of this study were similar to those of previous studies on the issue of respiratory infection prevention [17,32,36]. In addition, the findings of this study were similar to findings of past studies that examined the effectiveness of educational programs for the elderly [20,23,37,38]. In this study, significant differences in social capital existed between groups and across time periods. While social capital in the experimental group increased rapidly immediately after the intervention and was highest six months after the intervention, social capital continuously decreased in the control group. This may be attributable to the fact that participation in group activities reinforces social networks [39,40]. In this study, the groups formed during participation in the program may have continued after the program ended, ultimately forming long-lasting social networks. Social capital may be also affected by the environments in which trainees’ knowledge can be shared.

The strength of this study is that a reinforcement session was provided to the trainees and the effects of such reinforcement session was also evaluated with follow-up surveys. For the surveys, knowledge, attitudes, practices, and social capital were all assessed with respect to the extent to which they had been increased after the reinforcement. Although decreases were found in the trainees’ attitudes, practices, and social capital between posttest and 1-month follow-up, increases in their knowledge, attitudes, practices, and social capital during the intervention period were even more increased between 1- and 6-month follow-ups. It would appear that the reinforcement was effective. The finding is similar to the study conducted by Nahm et al. [22] in which a hip fracture prevention website program was developed to educate the elderly and the participants of the study were allowed to use the website autonomously for three months after the program ended. The result of a 3-month follow-up test showed that the participants had maintained their knowledge, self-efficacy, outcome expectancy, and behavior for three months after the intervention. A similar result was also found in a weight loss exercise program for the elderly [41]. The study group was asked to participate in a one-hour session once a week for up to 6 months. The frequency of the educational sessions was then reduced twice a month for 12 months, then once a month up to 18 months. The effect of the program was evaluated 6 months and 18 months after the intervention. The program was shown to have improved the quality of life of the elderly participants by facilitating enhanced self-efficacy and weight loss across the intervention period. In addition, Williams et al. [42] developed a program for smoking cessation based on self-determination theory. In this study, four additional sessions were provided to the participants for six months after the program ended, and it was suggested that these sessions may have helped the participants to maintain smoking cessation. An interesting finding was found in the study of Arola et al. [43]. They developed a health promotion program and applied it to a migrant elderly population on the basis of the Sautogenic approach. Similar to our study, this study showed a significant improvement in the sense of coherence—the ability to cope with stressful situations in daily life—by 6 months after the intervention. However, the effect disappeared in a 12-month follow-up without any additional intervention. Considering that it takes about 6 months to five years to internalize behavior change [44], a one-time intervention might be insufficient to trigger the long-term effects. Thus, interventions should be continued for at least 6 months in order to maintain a program’s long-term effectiveness.

A limitation of this study is that the sample only included elderly residents from rural areas near a small city. Consequently, the findings might not be generalizable outside this population, and thus studies on elderly populations living in remote areas or living in isolation, as well as elderly populations in other countries, are needed. Additionally, longitudinal research occurring over a period of at least one year is required to confirm the long-term effects of the respiratory infection prevention program. It would also be useful to perform research on other topics, such as nutrition and environmental management, within the context of respiratory infection prevention programs. Nonetheless, this study was meaningful given that the same group participated in the program for six months, and the short-term and long-term effects of the program were evaluated through repeated measures over this period of time.

## 5. Conclusions

Based on social cognitive theory (SCT), we developed an educational program for respiratory infection prevention for rural elderly residents in South Korea. The effectiveness of the program was investigated in terms of knowledge, attitudes, and practices about respiratory infection prevention, as well as social capital. The results showed that knowledge, attitudes, and practices for preventing respiratory infections were improved among the elderly residents who participated in the educational program. Social capital in the experimental group increased over time, whereas it decreased in the control group. In particular, differences between experimental and control groups over time periods were greater in practices than in knowledge and attitudes, which indicates that the educational program is a highly practical program. In addition, the program remained effective one month after the intervention, but a reinforcement session extended the program’s effects up to six months later. However, the six-month follow-up after the intervention may not be enough to conclude that this intervention has long-term effectiveness. Therefore, additional follow-ups, such as at one and two years after the intervention, are required in future studies. The effectiveness of the educational program also needs to be evaluated not only through subjective measures but also through objective measures (e.g., objective knowledge about respiratory infection prevention, frequency of respiratory infection prevention practices). Built on these academic efforts, it is expected that our educational program can be used as an effective intervention, one which can help rural elderly residents prevent respiratory infections. 

## Figures and Tables

**Table 1:** Study Design.

	Rural Elderly Residents					
	Pretest	Tx. 1 ^a^	Posttest	Follow-Up 1 ^b^	Tx. 2 ^c^	Follow-Up 2 ^d^
Exp. group	Y	Y	Y	Y	Y	Y
Cont. group	Y	-	Y	Y	-	Y

^a^ Tx. 1: Exposure to the health education program for respiratory infection prevention; ^b^ Follow-up 1: 1 month after Tx. 1; ^c^ Tx. 2: Exposure to the reinforcement program; ^d^ Follow-up 2: 6 months after Tx. 1.

**Table 2:** Contents of the RIPEP-SCT.

Theoretical Concept	PROGRAM				
	Session 1	Session 2	Session 3	Session 4	Session 5
	Cough Etiquette	Correct Hand Washing	Correct Toothbrushing	Correct Walking	Reinforcement Training
Attention stage	•Introduction to the purpose of the program • Need for prevention of respiratory infections •Understanding the trainees’ thoughts about respiratory infections	•Past session review •Singing a song: cough etiquette •Quiz about what the trainees learned in the past session •Experience sharing •Questions about today’s educational topics •Hand washing status (frequency, procedure)	•Past session review •Singing a song: cough etiquette, hand washing •Quiz about what the trainees learned in the past session •Experience sharing •Questions about today’s educational topics •Toothbrushing status (frequency, procedure) •Dry mouth experience	• Past session review •Singing a song: cough etiquette, hand washing, toothbrushing, oral routine •Quiz about what the trainees learned in the past session •Experience sharing •Questions about today’s educational topics	•Greeting •Singing a song: cough etiquette, hand washing, toothbrushing, oral routine, walking •A pledge of practice for preventing respiratory infections
Memory Stage	•Providing information on the nature and prevention of respiratory infections •Importance of cough etiquette •Guidance on how to wear a mask correctly •Video material for cough etiquette	•Importance of hand washing •Six steps of hand washing •Video material for hand washing	•Importance of oral hygiene • Preventing dry mouth •Correct toothbrushing demonstration (using dental model) •Video materials for oral routine	•Importance of walking •Effects of walking •Things to keep in mind when walking •Video materials for walking	•General questions •Singing a song: respiratory infection prevention •Presenting experiences on prevention of respiratory infections •Questions about lessons learned •Team game, true/false quiz •Finding the keywords about lessons learned
RetentionStage	•Mask wearing demonstration and practice	•Six steps of hand washing demonstration and practice	•Correct toothbrushing experience •Oral routine experience for saliva promotion	•Correct walking demonstration •Correct walking experience	
MotivationStage	•Singing a song: cough etiquette and mask wearing •A pledge of practice •Practicing cough etiquette and correct mask wearing and sharing information with neighbors	•Singing a song: correct hand washing •A pledge of practice •Practicing correct hand washing and sharing information with neighbors •Singing a song: cough etiquette, hand washing	•Singing a song: correct toothbrushing, oral routine •Practicing correct toothbrushing and oral routine, and sharing information with neighbors	•Singing a song: correct walking •Practicing correct walking and sharing information with neighbors •Singing songs that the trainees created over the four sessions	•Practice for preventing respiratory infections •Singing a song about respiratory infection prevention

**Table 3:** Results of homogeneity testing (*N* = 69).

Characteristics	Categories	Exp. (*n* = 37)	Cont. (*n* = 32)	*x*^2^/t	*p*
		*n* (%)/M ± SD	*n* (%)/M ± SD		
Gender	Male	7 (18.9)	10 (31.3)	1.41	0.236
	Female	30 (81.1)	22 (68.8)		
Age		76.62 ± 5.13	74.38 ± 5.70	1.99	0.089
Family type	Alone	11 (29.7)	11 (34.4)	0.17	0.680
	With family (sons, daughters, grandsons or granddaughters)	26 (70.3)	21 (65.6)		
Education	Uneducated (including elementary school dropouts)	18 (48.6)	9 (28.1)	3.04	0.219
	Elementary school graduate	13 (35.1)	16 (50.0)		
	Middle school graduate and over	6 (16.2)	7 (21.9)		
Subjective health status	Not healthy	9 (24.3)	9 (28.1)	0.19	0.909
	Ordinary	18 (48.6)	14 (43.8)		
	Healthy	10 (27.0)	9 (28.1)		
Frequency of watching health-related programs	≤1 Tw ^d^	5 (13.5)	4 (12.5)	5.41	0.176
	2–3 Tw	8 (21.6)	12 (37.5)		
	≥4 Tw	15 (40.5)	14 (43.8)		
	Every day	9 (24.3)	2 (6.2)		
Sources of health information (multiple replies allowed)	News, TV, radio,	32 (71.1)	18 (54.5)		
	Community health posts or health centers, hospitals	41 (91.1)	28 (84.8)		
	Family, friends, neighbors	10 (22.2)	1 (3.0)		
	Others (internet, smartphone, etc.)	5 (11.1)	2 (6.1)		
Attending cough etiquette education program ^a^	yes	3 (8.1)	8 (25.0)	3.65	0.056
	no	(91.9)	24 (75.0)		
Attending hand washing education program ^b^	yes	3 (28.9)	13 (39.4)	1.43	0.232
	no	32 (71.1)	20 (60.6)		
Chronic disease	No disease	8 (17.8)	8 (24.2)		
	Hypertension	30 (66.7)	20 (60.6)		
	Diabetes	10 (22.2)	10 (30.3)		
	Cardiac diseases	5 (11.1)	9 (27.3)		
	Hyperlipidemia	4 (8.9)	4 (12.1)		
	Joint diseases	8 (17.4)	1 (3.0)		
	Others	10 (22.2)	1 (3.0)		
Knowledge of RIP ^c^		9.11 ± 1.29	8.84 ± 1.14	0.90	0.373
Attitude of RIP		4.44 ± 0.43	4.70 ± 0.36	−2.70	0.009
Practice of RIP		3.71 ± 0.51	3.94 ± 0.54	−1.78	0.080
Social capital		3.80 ± 0.74	3.95 ± 0.47	−0.97	0.335

^a^ Fisher’s exact test; ^b^ Multiple responses; ^c^ Respiratory infection prevention; ^d^ Tw: Times per week.

**Table 4:** Descriptive statistics for dependent variables and results of mixed ANOVA analyses (*N* = 69).

Variable	Group	T0 ^d^	T1 ^e^	T2 ^f^	T3 ^g^	Source	*F*	*p*	$\eta_{p}^{2}$
		M ± SD	M ± SD	M ± SD	M ± SD				
Knowledge about RIP ^a^	Exp.(*n* = 37) ^b^	0.91 ± 0.13	0.96 ± 0.05	0.97 ± 0.06	0.98 ± 0.04	G ^h^	7.34	0.009	0.10
	Cont.(*n* = 32) ^c^	0.88 ± 0.11	0.91 ± 0.10	0.97 ± 0.06	0.91 ± 0.13	T ^i^	9.50	<0.001	0.12
						G × T ^j^	3.09	0.038	0.04
Attitudes toward RIP	Exp.(*n* = 37)	4.44 ± 0.43	4.82 ± 0.32	4.59 ± 0.43	4.94 ± 0.13	G	0.12	0.734	0.00
	Cont.(*n* = 32)	4.70 ± 0.36	4.61 ± 0.34	4.65 ± 0.33	4.77 ± 0.36	T	10.03	<0.001	0.13
						G × T	8.03	<0.001	0.11
RIP practices	Exp.(*n* = 37)	3.71 ± 0.51	4.46 ± 0.43	4.31 ± 0.48	4.58 ± 0.29	G	20.56	<0.001	0.24
	Cont.(*n* = 32)	3.94 ± 0.54	4.07 ± 0.48	3.84 ± 0.38	3.98 ± 0.44	T	18.85	<0.001	0.22
						G × T	14.10	<0.001	0.17
Social Capital	Exp.(*n* = 37)	3.81 ± 0.74	4.23 ± 0.46	4.21 ± 0.43	4.39 ± 0.33	G	17.95	<0.001	0.21
	Cont.(*n* = 32)	3.95 ± 0.47	3.95 ± 0.47	3.82 ± 0.37	3.72 ± 0.35	T	3.17	0.032	0.05
						G × T	10.81	<0.001	0.14

^a^ RIP: Respiratory infection prevention; ^b^ Exp.: Experimental group; ^c^ Cont.: Control group; ^d^ T0: Pretest; ^e^ T1: Posttest; ^f^ T2: 1-month follow-up; ^g^ T3: 6-month follow-up; ^h^ G: Group; ^i^ T: Time; ^j^ G × T: Group × Time.
